# On Dilitation of the Os Uteri during Labor by Incisions

**Published:** 1866-07

**Authors:** 

**Affiliations:** Konigsburg


ON DILITATION OF THE OS UTERI DURING LABOR BY INCISIONS.—Dr. Hilderbrandt, of Konigsburg.—The author commences by a brief account of nine labors, in seven of which primiparæ, advanced in life, suffered from rigidity of the os uteri; against which ipecacuanha, opium, poultices, baths, bleedings, and chloroform were all unavailing.

Incisions were made, after which all the cases were fortunately terminated.

Incisions were also made, with a like favorable result, in one case of convulsions, and one in prolapsus of the cord. He proceeds to consider the proposed risks that have deterred accoucheurs from the performance of the operation. It has been feared that the pain of incisions, in a part already irritated by a fœtal pressure, and in persons inclined to nervous disorder by prolonged labor, might be productive of mischief. This fear is wholly groundless; the incisions themselves being scarcely felt by the patient, and the relief actually afforded by them being very great. Others have dreaded an extension of the incisions during pain, so that they might come to involve the substance of the uterus, and produce the fatal effects of rupture. This is visionary. The incisions do sometimes yield a little, but never so far as to reach even the cervical portion of the womb; and the operator, by relieving an impediment to the advance of the fœtus, diminishes instead of increases the danger of rupture.

Lately, it has been feared that excessive hemorrhage might attend or follow the incisions, but this fear is never realized in practice. In cases that require such treatment, the os uteri is morbidly changed, and so bloodless that the hemorrhage from the excisions does not exceed a few drops. Where incisions are made into a healthy uterus, in order to effect a rapid delivery, the bleeding may be greater, but its source is always accessible, and it may, therefore, always be readily controlled, while in such cases, which are almost limited to eclampia and placenta prævia, the danger from hemorrhage can never be equal to the danger of delay. The operation is chiefly indicated, however, in morbid conditions of the vaginal portion of the cervex, such as rigidity, hypertrophy, and malignant disease. For forced delivery, with a healthy cervix, the incisions should be six or eight in number, and not more than three lines in depth.—*N. Y. Jour. of Medicine.*

